# Genome Analysis of A Novel Recombinant Human Adenovirus Type 1 in China

**DOI:** 10.1038/s41598-018-37756-4

**Published:** 2019-03-12

**Authors:** Wanju Zhang, Lisu Huang

**Affiliations:** 1 Shanghai Public Health Clinical Center, Fudan university, Shanghai, 201508 China; 2 Xinhua Hospital, Shanghai Jiao Tong University School of Medicine, Shanghai, 200092 China

## Abstract

Human adenovirus (HAdV) group C are the common etiologic in infants with severe acute respiratory infections (SARI). In the study, we report that a novel recombinant HAdV-C group strain (SH2016) was isolated from an infant with SARI in Shanghai in Feb. 4, 2016. The whole-genome sequence of SH2016 strain was generated and compared to other HAdV genomes publicly available. The strain SH2016 genome contains 35,946 nucleotides and coded 40 putative proteins, which was divided into 11 regions. RDP and phylogenetic analyses of the complete genome showed that the SH2016 strain was arranged into a novel subtype and might be recombined with HAdV-1 and HAdV-2. Our finding indicated that the frequent recombination among the HAdV-C group played an important role in driving force for polymorphism of human HAdV-C group prevalent in Shanghai, China. Further epidemiological surveillance of HAdV-C group is necessary to explore whether the novel HAdV-C group will maintain long-term stability. And the pathogenicity and clinical characteristics of the novel HAdV-C group member should be done more.

## Introduction

The international committee on taxonomy of viruses had divided Adenoviridae into 5 genera, Atadenovirus, Aviadenovirus, Mastadenovirus, Siadenovirus, and Ichtadenovirus. Through more than 6 decades, since the first characterizations of human adenoviruses (HAdVs)^1^, all of HAdVs falled within the genus Mastadenovirus. And HAdVs were classified into 7 groups (HAdV-A to HAdV-G), including 52 serotypes and 90 human HAdV genotypes^2–6^, which were recognized by Human Adenovirus Working Group, July, 2018 Update (http://hadvwg.gmu.edu/). Over the past 30 years, recombinant adenovirus-vectors based on the HAdV-C group had also been developed and extensively used in preclinical and clinical studies^7^. Among of these, members of the HAdV-B group (types 3, 7, 11, 14, 16, 21, 34, 35, 50 and 55) and HAdV-C group (types 1, 2, 5, 6 and 57) cause a variety of typically acute respiratory diseases. Especially, HAdV-C group could cause severe bronchiolitis or pneumonia in the early childhood^8,9^.

Three recombinant HAdV-C strains (BJ04, BJ09 and CBJ113), classified within HAdV-2 (P1H2F2), isolated from infants with acute respiratory infection in Beijing in 2009–2013 by labs in China CDC^10,11^. Among three strains, CBJ113 was characterized by a recombination among HAdV-2, HAdV-6, HAdV-1, HAdV-5, and HAdV-57 sequences. BJ04 recombination event involved parental strains HAdV-1, HAdV-2, whereas BJ09 involved in HAdV-1, HAdV-5 and CBJ113. Therefore, HAdV-1 was involved in recombination of other HAdV-C types.

The National Adenovirus Type Reporting System (NATRS) of the United States described trends in reported HAdV-C group was circulating in the United States after initiation of surveillance in 2014^8^. NATRS also displayed that HAdV-1 was identified as the pathogen responsible for that outbreak^8^. Interestingly, here, we describe the characterization of a novel type of HAdV-1 isolated from a hospitalized infant with SARI. We found that there was the possibility of intraspecies recombination among HAdV-C group on the whole genome sequence analysis. In order to gain a better understanding of this phenomenon, we determined and analyzed the whole-genome sequence of HAdV-1 strain SH2016.

## Results

### Isolation and complete genomic characterization of the novel HAdV-C type

**Figure 1:**
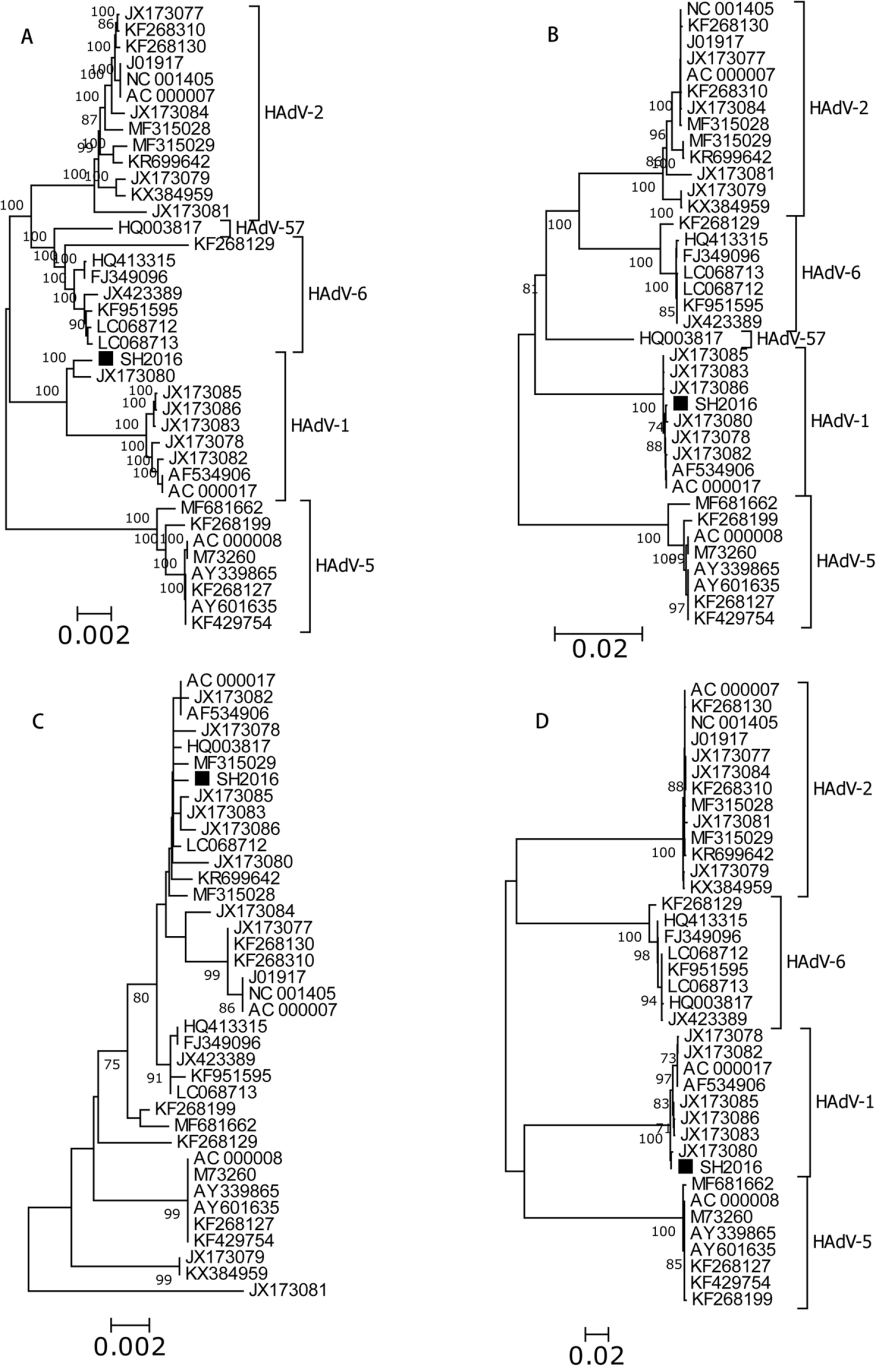
Neighbor-joining phylogenetic trees based on the open reading frame sequences of the whole genome (**A**), hexon gene (**B**), penton gene (**C**) and fiber gene (**D**) of SH2016 strain in this study and those of HAdV-C whole genome reference strains from GenBank. Strain SH2016 highlighted with a solid square was characterized in this study. The trees were constructed using the neighbor-joining method of MEGA 6.06 with 1000 bootstrap trials performed to assign confidence to the grouping.

Throat swabs positive for the HAdV, when other viral nucleic acid detection was negative, were used initially for viral isolation. The isolated strain caused a visible CPE on culturing. It was archived as strain “human/China/SH/2016/1[P1H1F1]”, which we referred to as “SH2016” strain. Using next-generation and Sanger sequencing, the full-length genomic sequence of strain SH2016 was determined, and the genomic data, was deposited in GenBank (accession number: MH183293). The genome length of SH2016 strain (35,946 bp) was similar to the length of the prototype strain, human adenovirus type 1 (AC_000017, 36001 bp). The G + C content of the genome was 55.2%, which is similar to G + C content of other members of species group C adenoviruses^1^, and the plus strand had an overall base composition of 23.23% A, 27.99% C, 27.21% G, and 21.57% T. Similar to the genomes of prototype HAdV-1 reference strain (AC_000017), the genome encoded 40 coding sequences (Table 1) and 35 non-coding motifs (Table 2) were recognized. Whole genome phylogenetic analysis of 43 archived complete HAdV genomes from GenBank illustrated that strain SH2016 were clustered into HAdV-1, but it branched out independently with human/EGY/E13/2001/1[P1H1F1]^12^ (Fig. 1A). Then, phylogenetic analysis of 3 major antigen genes (penton, hexon and fiber) of the SH2016 showed that the 3 genes were classified to H1, P1, and F1 (Fig. 1B–D).

**Table 1:** Genome annotation of SH2016.

Gene	Product	Location	No. of amino residues
E1A	cds	560–1,546	NT
	E1A 29 kDa	560–1,112, 1,230–1,546	289
	E1A 26 kDa	560–974, 1,230–1,546	243
	E1A 6 kDa	560–637, 1,230–1,546	55
E1B	cds	1,717–3,515	NT
	E1B 19 kDa	1,717–2,250	177
	E1B 55 kDa	2,022–3,515	497
pIX	pIX 14 kDa	3,613–4,035	142
Iva2 C	Iva2 51 kDa	4,094–5,430, 5,709–5,721	449
E2B C	E2B pol	5,200–8,787, 14,117–14,125	1198
L1	L1 13.6 kDa	7,981–8,430	149
pTP C	E2B pTP	8,586–10,592, 14,117–14,125	671
L1	L1 52 kDa	11,053–12,300	415
	pIIIa	12,321–14,078	585
L2	Penton	14,162–15,886	574
	pVII	15,893–16,489	198
	pV	16,559–17,665	368
	pX	17,693–17,935	80
L3	pVI	18,019–18,771	250
	Hexon	18,858–21,751	964
	Protease 23 kDa	21,785–22,399	204
E2A C	DBP	22,497–24,086	529
L4	cds	24,115–27,905	NT
	Hexon-assembly protein 33 kDa	24,115–26,538	807
	Spicing factor 33 kDa	26,249–26,564, 26,767–27,134	227
	Encapsidation protein 22 kDa	26,249–26,833	194
	pVIII	27,222–27,905	227
E3	cds	27,906–30,842	
	Immune modulating protein 12.5 kDa	27,906–28,229	107
	CR1-alpha	28,642–28,827	61
	Immune modulating protein 19 kDa	28,824–29,303	159
	CR1-beta	29,480–29,785	101
	RID-alpha	29,793–30,068	91
	RID-beta	30,071–30,643	130
	Control protein 14.7 kDa	30,456–30,842	128
	Putative protein U C	30,865–31,032	56
L5	Fiber	31,043–32,791	582
E4	cds	32,920–35,537	
	Control protein orf 6/7 C	32,920–33,198, 33,910–34,083	150
	Control protein orf 5 (34 kDa) C	33,199–34083	294
	Control protein orf 3/4 C	34,004–34,088, 34,614–34,714	61
	Control protein orf 4 C	34,004–34,348	114
	Control protein orf 3 C	34,364–34,714	116
	Control protein orf 2 C	34,711–35,103	130
	Control protein orf 1 C	35,151–35,537	128

The complementary strand functions are marked as ‘c’, for example Iva2c.

**Table 2:** SH2016 genome non-coding motifs annotations.

Motif (5′ to 3′)	Function	Nucleotide locatity
CATCAT…CGTAG	ITR	1–103
AATAATATACC	DNApol-pTP-binding site	8–18
TATGATAATGA	NFIII-binding site	39–49
GGGGGTGG	Sp1-recognition	50–56
TGACGT	Transcription factor ATF-binding site	64–69
GGGCGTGG	Transcription factor Sp1-recognition site	76–83
CGGGGCGG	Transcription factor Sp1-recognition site	87–92
TGACGT	Transcription factor ATF-binding site	96–101
TAAATA	TATA box for E1A	468–473
AATAAA	polyA signal for E1A	1,612–1,617
TATATA	TATA box for E1B	1,675–1,680
ACTGAA	polyA signal for E1B	3,519–3,525
TATATAA	TATA box for pIX gene	3,557–,3563
AATAAA	polyA signal for pIX gene	4,042–4,047
AATACA	polyA signal for E2B	4,081–4,086 C
TGATTGGTTT	Inverted CAAT box for MLP	5,970–5,979
GCCACGTGAC	Upstream element for MLP	5,990–5,999
GAAGGGGGGG	MAZ/Sp1-binding site for MLP	6,011–6,020
TATAAAA	TATA box for MLP	6,021–6,027
GGGGGTGGGGG	MAZ/SP1-binding site for MLP	6,028–6,038
TCACTCT	Initiator element for MLP	6,050–6,056
TTGTCAGTTTCCA	DE1 for MLP	6,137–6,149
AACGAGGAGGATTTGA	DE2a & DE2b for MLP	6,152–6,167
AATAAA	polyA signal for L1	14,103–14,109
ATTAAA	polyA signal for VII gene	16,495–16,500
AATAAA	polyA signal for V gene	17,967–17,972
AATAAA	polyA signal for L3	22,425–22,430
TATAAC	TATA box for E3	27,587–27,592
AACACA	polyA signal for L4	27,938–27,943
AATAAA	polyA signal for E3	30,844–30,849
AAAAAA	polyA signal for L5	32,837–32,842
AATATA	polyA signal for E4	32,947–32,952 C
TATATATA	TATA box for E4	35,643–35,650 C
ATAATATACC	DNApol-pTP-binding site	35,929–35,938 C
CATCAT…CGTAG	Inverted terminal repeat	35,844–35,946 C

ITR: Inverted terminal repeat, DNApol-pTP-binding site: the pre-terminal protein-DNA polymerase complex binding site, NFIII: nuclear factor III, MLP: major-late promoter, DE1: Downstream sequence element 1, DE 2a & DE2b: Downstream sequence element 2a & 2b, MAZ: Myc-associated Zinc Finger Protein.

DNA non-coding sequence motifs are recognized for the novel Shanghai HAdV-1 strain (human/CHN/SH/2016/1[P1H1F1]). The nucleotide signatures and their putative functions are indicated. The complementary strand functions are marked as ‘c’, for example 4081–4086c.

### Comparative genome analysis

**Figure 2:**
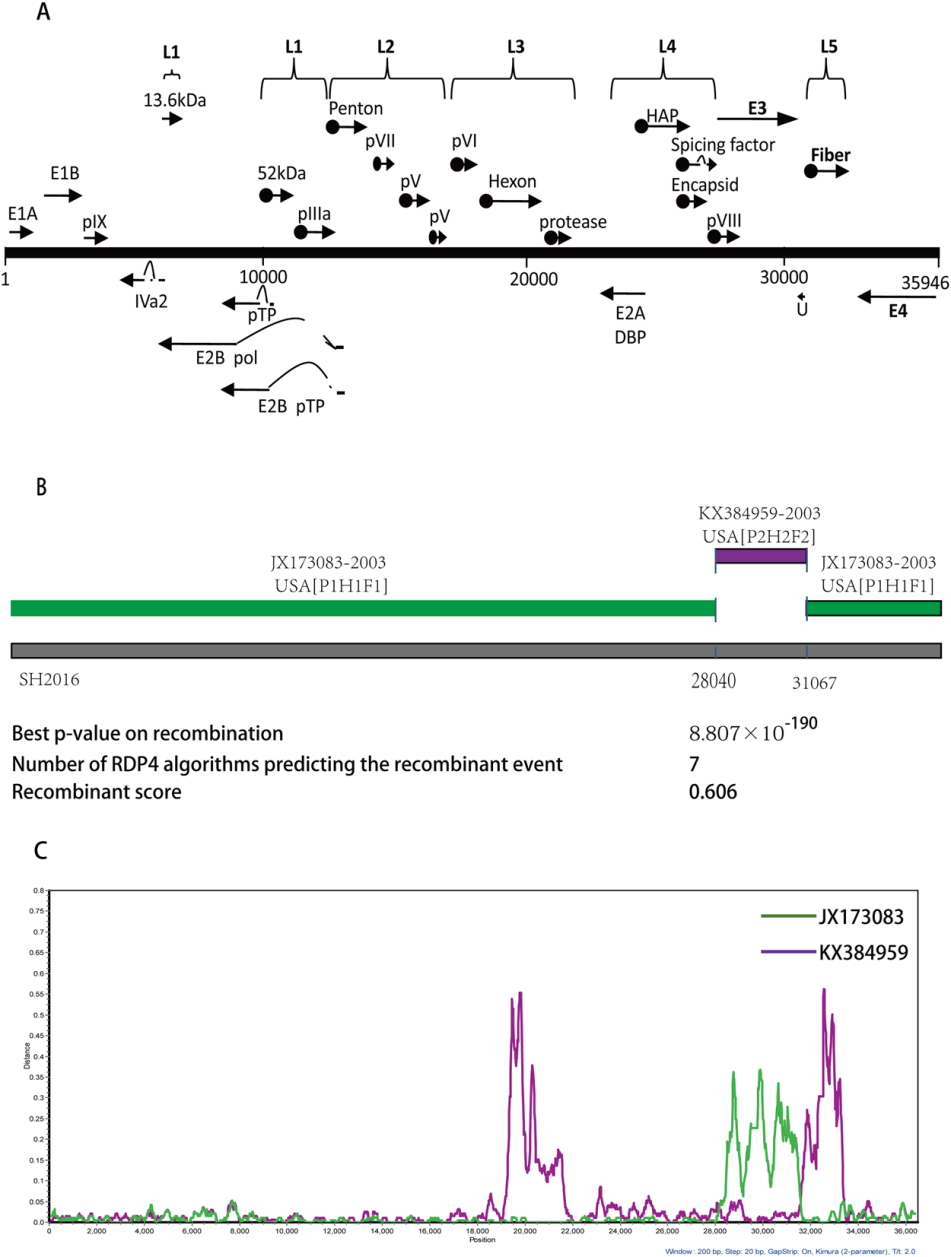
Genetic recombinant analyses of the complete genome of the novel strain SH2016. (**A**) Genomic map of strain SH2016. The l-strand of the genome is represented by a straight line. Rightward (top) and leftward (bottom) ORFs are represented by grey arrows. (**B**) Recombination events predicted in strain SH2016. Strain SH2016 genome is shown as a thick black line. The likely backbone is shown as a cyan line. Genetic components predicted by RDP4 to be involved in a recombination event are shown as purple line. Likely breakpoint positions are shown below the genome. (**C**) Similarity analyses of SH2016. SH2016 was used as the query sequence to compare with other 2 representative strains of HAdV-C. The default setting of SimPlot software was used as followed: Window size 200 bp, step size 20 bp, replicates 1000 times and tree model neighbor-joining.

Compared with the complete group C genome sequences of the 5 prototype strains of HAdV-1 (AC_000017), HAdV-2 (AC_000007), HAdV-5 (AC_000008), HAdV-6 (HQ413315) and HAdV-57 (HQ003817), the SH2016 strain is conserved, sharing the highest nucleotide identity (97.93%) with the prototype strain of HAdV-1 (Table 3). Based on the nucleotide alignment of the different gene sequences, the nucleotide sequences of the penton, hexon and fiber genes showed the highest degree of homology between the prototype strain HAdV-1, with identities of 99.82%, 99.68% and 98.79% respectively. Genomic map of strain SH2016, contained 40 ORFs (rightward ORFs: 33, leftward ORFs: 7), was showed in Fig. 2A. Comparison of the nucleotide sequences of the 11 coding regions (E1A, E1B, E2B, L1 13.6 kDa, pTP, L1 52 kDa, pIIIa, pVII, E3 and E4) showed the highest sequence similarity between strains HAdV-2, HAdV-5, HAdV-6, and HAdV-57, with identities of 98.19~99.75%. On the other hand, HAdV-1 and HAdV-57 showed the greatest similarities to SH2016 in the pIX gene (99.53%), HAdV-6 and HAdV-57 in putative protein U gene (98.19%), HAdV-1, HAdV-5 and HAdV-6 in pX gene (99.59%), respectively. While the Iva2, pV, pX, pVI, DBP, and L4 coding regions displayed the highest similarity with HAdV-1.Through comparative genomics analysis, the novel HAdV-1 type showed limited sequence variation between the HAdV-C group.

**Table 3:** The nucleotides sequence identities between SH2016 and HAdV-C reprensentative strains.

Region	% nucleotide identities of Novel-HAdV-1				
	HAdV-1	HAdV-2	HAdV-5	HAdV-6	HAdV-57
5′-Terminal (no ITR)	98.44%	98.45%	98.00%	99.12%	99.12%
E1A	98.56%	98.77%	99.28%	98.87%	98.77%
E1B	99.21%	99.27%	98.58%	99.16%	99.44%
pIX	99.53%	99.05%	99.29%	99.05%	99.53%
Iva2	98.88%	98.58%	98.65%	98.58%	98.81%
E2B	98.11%	98.34%	98.51%	98.42%	98.23%
L1 13.6 kDa	99.33%	99.10%	99.11%	99.55%	99.33%
pTP	99.10%	99.15%	99.75%	99.30%	99.10%
L1 52 kDa	99.11%	99.60%	98.46%	99.44%	99.11%
pIIIa	99.31%	99.43%	98.85%	99.03%	99.14%
Penton	99.82%	99.18%	98.41%	99.59%	99.82%
pVII	99.16%	98.30%	98.65%	99.33%	99.16%
pV	99.73%	98.54%	97.89%	99.00%	99.36%
pX	99.59%	99.59%	99.59%	99.17%	99.17%
pVI	99.87%	97.70%	98.79%	96.59%	96.45%
Hexon	99.68%	84.48%	82.34%	83.16%	87.88%
Protease 23 kDa	99.18%	98.02%	97.36%	99.51%	97.69%
DBP	99.49%	96.85%	96.38%	96.72%	96.52%
L4	99.60%	98.53%	97.25%	98.56%	97.71%
E3	82.79%	98.39%	79.54%	98.20%	98.31%
Putative protein U	85.50%	96.33%	83.10%	98.19%	98.19%
Fiber	98.79%	66.08%	69.10%	72.90%	72.69%
E4	98.69%	98.80%	98.41%	98.92%	98.45%
3′-Terminal (no ITR)	98.30%	97.96%	98.65%	97.95%	97.96%
Complete genome	97.93%	96.44%	94.66%	96.62%	96.78%

### Genomic recombination analysis of strain SH2016

**Figure 3:**
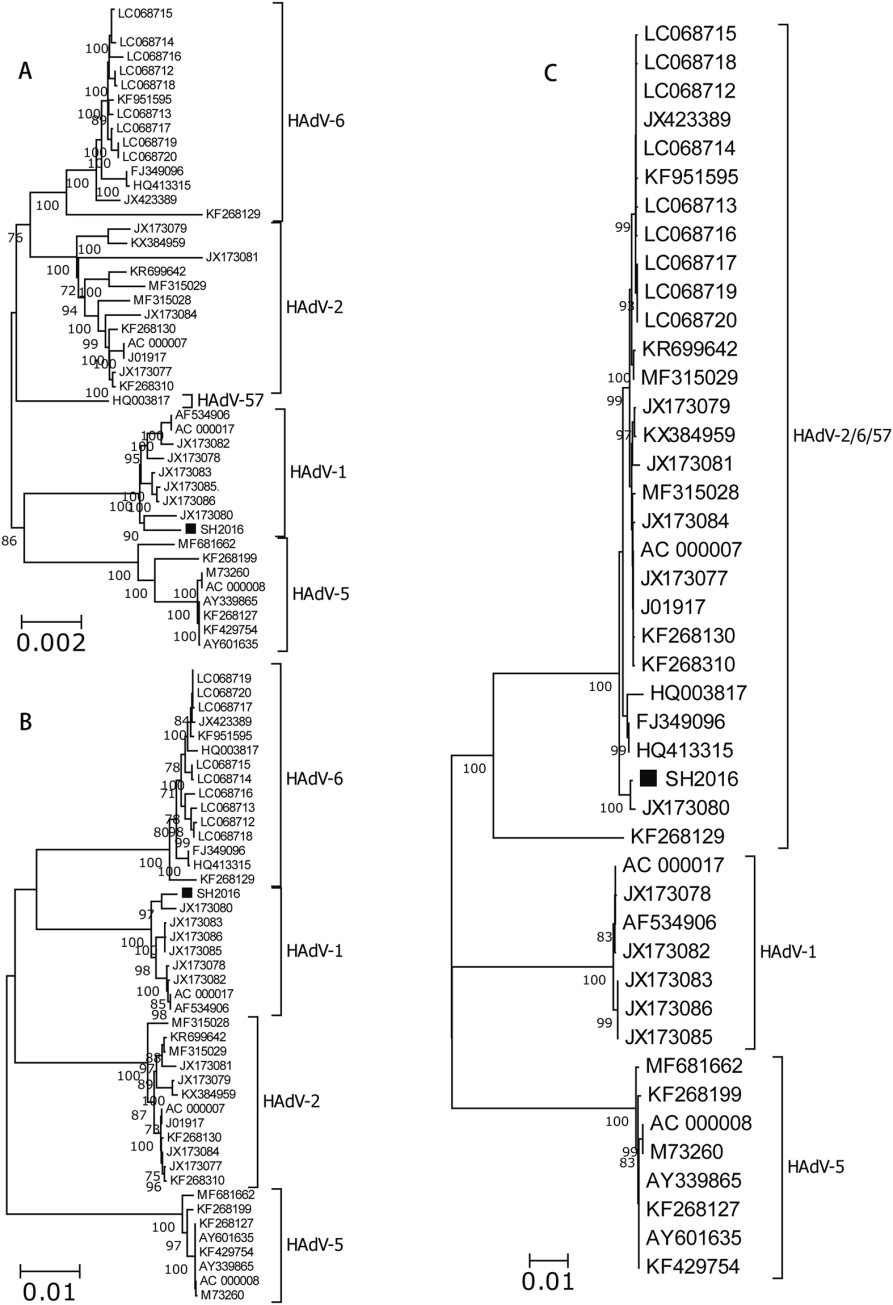
Neighbor-joining phylogenetic trees based on the left region (**A**) of recombinant point (5′-end), the right region (**B**) of recombinant point (3′-end) and the recombinant frame (**C**) sequences of the recombinant regionof SH2016 strain in this study and those of HAdV-C whole genome reference strains from GenBank. Strain SH2016 highlighted with a solid square was characterized in this study. The trees were constructed using the neighbor-joining method of MEGA 6.06 with 1000 bootstrap trials performed to assign confidence to the grouping.

RDP4 package strongly predicted that the strain SH2016 was a highly probable homologous recombinant resulting from HAdV-1 (strain: human/USA/VT2672/2003/1[P1H1F1], GenBank ID: JX173083) and HAdV-2 (stain: T215/Ft Jackson South Carolina USA/2002, GenBank ID: KX384959) with beginning breakpoint located around 28040 (without gaps) of HAdV-1, within the gene coding for putative host modulation protein E3 (early E3 12.5 kDa glycoprotein) and with ending breakpoint located around 31067 (without gaps) of HAdV-1, within the gene coding for fiber protein (Fig. 2B). The similarities with possible major parent strain (HAdV-1) and minor parent strain (HAdV-2) were 99.3% and 98.6%, respectively. Indeed, 7 algorithms (RDP, GENECONV, BootScan, MaxChi, Chimaera, SiScan, 3Seq, LARD, PhylPro (Supplemental Figs S1–7), were utilized to predict potential recombination events between the input sequences) supported this event with p-values ranging from 2.347 × 10^−187^ to 2.179 × 10^−12^ (Table 4). Similarity plot analysis using SimPlot software were performed to confirm the consequent of recombination events within the genome of SH2016. As well as, SimPlot analysis indicated that the mosaic structure comprised of the SH2016 genome originated not only from mainly circulating viral strain: prototype HAdV-1 basically, but also from a small quantity of HAdV-2 (Fig. 2C). The results coincided with phylogenetic analyses, which indicated that both of the left region of recombinant point (5′-end, 1–28039) and the right region of recombinant point (3′-end, 31067–35946) of SH2016 strain were clustered into HAdV-1 group with high confidence (bootstrap value = 100% or 97%, Fig. 3A,B), but the recombinant region was clustered into HAdV-2/6/57 group (bootstrap value = 100%, Fig. 3C). So these findings re-confirmed that SH2016 appeared from potential genetic recombination events, which HAdV-1, and HAdV-2 participated in this process.

**Table 4:** The number of algorithms of the RDP4 package that were predicting the recombination event and recombinant score are shown.

Recombinant strain	Parent major/minor	Recombinant region in alignment	Model (average p-value)							Recombinant score
			RDP	GENECONV	Bootscan	MaxChi	Chimaera	Siscan	3Seq	
SH2016	JX173083/KX384959	28185–31576	2.347 × 10^−187^	8.807 × 10^−190^	8.032 × 10^−172^	1.448 × 10^−49^	4.506 × 10^−51^	9.637 × 10^−59^	2.179 × 10^−12^	0.606

## Discussion

In order to ensure the accuracy of the results, the phylogenetic trees were also constructed by maximum likelihood (ML) method implemented in IQ-TREE 1.6.7.1^13^ under the most suitable nucleotide substitution models respectively, which were selected by jModeltest^14^ [Supplemental Figs S8–14]. The frameworks of all neighbor-joining (NJ) trees in this study were consistent with ML trees. Intriguingly, the penton NJ-tree was not really informative as the 43 sequences did not feature much divergence between each other (Fig. 1C), and the same situation still appeared in maximum likelihood tree (Fig. S10). This showed that SH2016 was convergent evolution with known HAdV-C sequences in the penton region. As the full genome trees had shown, SH2016 was related to AC_000017 which could be considered as the backbone of the prototype HAdV-1 genome (Fig. 1A, Supplemental Fig. S8). However, SH2016 genome sequence was showing some divergence at the E3 region and putative protein U region of the genome, both of which were located in reconstituted area. The E3 region and putative protein U region of the SH2016 genome (major areas of recombination) were more divergent than the rest of the genome, which had only 82.79% and 85.50% identities with the prototype HAdV-1 (Table 3), respectively.

In summary, the complete genome sequence of the novel recombinant HAdV-1 strain (SH2016) was determined and characterized, isolated in Shanghai, China. Phylogenetic and SimPlot analyses both displayed that the novel subtype of HAdV-1 (SH2016) was a recombinant event involving HAdV-1 and HAdV-2 (Figs 2B,C and 3). And The recombination area was located between 28040 and 31067, which including most of E3, whole U and few of L5 (Fig. 2A,B). However, the process of intratypic recombination incident is not clear in its evolutionary history, only in the case that more sequences were needed to investigate the spatiotemporal relationships of the novel HAdV-C group all over the world.

Comparison of the amino acid sequences of the fiber, hexon and penton of strain SH2016 with other type HAdV-1 fibers, hexons and pentons, only the fiber of strain SH2016 has three mutations. According to the protein structure, the fiber of SH2016 strain could also be divided into three components including an N-terminal tail (FNPVYPYD)^2,15,16^, two repeat/shaft regions and a C-terminal globular knob^17–20^. One (A71T) of mutations occurred in the first repeat/shaft region, and two (V432I and H470N) other mutations occurred in C-terminal globular knob, which is typically responsible for interaction with the cell receptors. Whether the mutations at these sites lead to antigenic drift need to be experimentally validated.

In conclusion, we propose that the SH2016 strain is a novel intratypic HAdV-C strain and may be an etiological agent of SARI. On the basis of their complete genome sequences, it arose through the recombination of two HAdV genotypes, HAdV-1 and HAdV-2, which frequently cause respiratory infection^9,21–23^. Whether the emergence of recombination strain might increase virulence, thereby posing a new global challenge with regard to acute respiratory diseases in the near future, warrants further investigation. So, epidemiological and virological surveillance of this uninvestigated respiratory disease pathogen should be strengthened.

## Material and Methods

### Specimen collection and identification

Throat swab specimens were collected from the outpatients with respiratory tract infection for surveillance subjects at designated intervals by trained medical staff of Xinhua Hospital Affiliated to Shanghai Jiao Tong University School of Medicine in this study. SH2016 was collected in February 4 at outpatient. The patient was more than two years old and clinically diagnosed with bronchitis and upper respiratory tract infection. The patient was diagnosed with human adenovirus infection and ruled out other possible common viral infections using our previous diagnostic methods^24^. After 3 days of antiviral treatment, the patient recovered.

### Cell culture and virus isolation

HEp-2 cells (from American Type Culture Collection, ATCC Number CCL-23, Manassas, VA, USA) were maintained in complete DMEM supplemented with 10% FBS, 100 U/mL penicillin, and 100 µg/mL streptomycin (Invitrogen, Carlsbad, CA, USA) at 37 °C with 5% CO2. For the virus culture, DMEM with 2% FBS and antibiotics was used. Cells inoculated with clinical samples, which were filtered by the 0.22 m filter (Millipore, Merch, Germany), were incubated at 37 °C for 7 days. If no cytopathic effect (CPE) was observed, the culture supernatants were used to inoculate fresh cells for 2 additional passages. And if the adenovirus-like CPE were appeared, the cultures were passaged again to confirm the presence of the viruses. Virus-infected cells and supernatant were collected and used for subsequent detection and genome sequencing.

### DNA extraction, PCR strategy and sequencing

Strain SH2016 was isolated from throat swab and underwent three passages in HEp-2 cells to obtain high-tilter stocks. The viral DNA was extracted using a QIAamp MinElute Virus Spin Kit (Qiagen, Germany) following the manufacturer’s instructions. The primer pairs (Supplementary Table 1) used to amplify complete genome was designed based primarily on of human mastadenovirus C strain CBJ113 (KR699642), human mastadenovirus C isolates human/CHN/BJ04/2012/[P1/H2/F2] (MF315028), human/CHN/BJ09/2012/[P1/H2/F2] (MF315029) and human adenovirus C strain human/EGY/E13/2001/1[P1H1F1] (JX173080)^12^, respectively.

Twenty four overlapping PCR fragments covering the entire genome were amplified by using the Platinum™ Taq DNA (Invitrogen, Thermo Fisher, CA, USA) according to the manufacturer’s protocol. PCR amplification was carried out at 95 °C for 5 min for one cycle to denature, and followed by 40 cycles for amplification at 95 °C for 30 s, 55 °C for 30 s, 72 °C for 180 s. At the end of the cycling, an additional extension period of 72 °C for 10 min was included, after which the samples were stored at 4 °C. For the 5′/3′-terminal genome sequences, the covalent junction between the purified DNA template and the terminal protein (TP) was broken by the addition of 0.4 N NaOH as described in Xu’s protocol^2^. The PCR products were separated by electrophoresis on 1.5% agarose gels and visualized under UV light. The PCR amplicon was then inserted into pGEM-T Easy Vector using TA cloning. The recombinant plasmid were identified by amplification primer pairs respectively, and was confirmed via sequencing using M13 forward and M13 reverse primers as sequencing primers. The recombinant plasmids were directly sequenced on an ABI 3730XL automatic DNA analyzer using an ABI Prism BigDye Terminator cycle sequencing kit 3.1 (Applied Biosystems). Either bracketing PCR or internal primers were used as sequencing primers to obtain overlapping and complementary sequences and a minimum twofold coverage. Whole genome sequences were obtained from 24 overlapping sequences assembled in ContigExpress Progect (Vector NTI).

### Nucleotide sequence accession number

Annotated genome sequence of SH2016 was submitted to GenBank database under the following accession number MH183293.

### Genome annotation

The BLASTn program (National Center for Biotechnology Information, Bethesda, MD, USA) was used to identify the homologous nucleotide sequences in the GenBank database (https://blast.ncbi.nlm.nih.gov/Blast.cgi?PROGRAM = blastn&PAGE_TYPE = BlastSearch&LINK_LOC = blasthome). The SH2016 genome sequence was annotated based on the previous annotation of HAdV-C strain (human/EGY/E13/2001/1[P1H1F1]). The DNA and protein sequence alignments were created by using BioEdit sequence alignment editor software (version BioEdit v7.1.3; Tom Hall, Ibis BioSciences, CA).

### Phylogenetic analysis

Phylogenetic trees were generated with MEGA6.06 using the neighbor-joining (NJ) method with the maximum composite likelihood nucleotide substitution model and bootstrap test of phylogeny with replicates set to 1000 to assign confidence to the grouping. The maximum likelihood (ML) phylogenetic tree were reconstructed by the ML method implemented in IQ-TREE 1.6.7.1^13^ based on the different models. Additional, the optimal evolutionary models were identified with the aid of the computer program jModelTest 2.1.7^14^. The resulting ML trees were created and edited using FigTree (http://tree.bio.ed.ac.uk/software/figtree/). Strain SH2016 highlighted with a solid square in NJ trees or with red fond in ML trees were characterized in this study, respectively.

### Recombination analysis

The aligned sequences of the SH2016 sequence available from GenBank were subjected to recombination analysis. The Recombination Detection Program (RDP) package Beta 4.96 was used for identifcation of recombinant sequences. Multiple methods in its default mode, such as RDP, GENECONV, BootScan, MaxChi, Chimaera, SiScan, 3Seq, LARD, PhylPro, were utilized to predict potential recombination events between the input sequences. Only those recombination events were taken into considerations which were supported by at least 4 methods to avoid misidentifcation using only a single methodology. The best signals for recombination are associated with the lowest P-values; the highest acceptable P-value was set to 0.05. Recombination events detected with RDP Beta 4.96 were confirmed and visualized with SimPlot Version 3.5.1. Bootscan analysis in the SimPlot package version 3.5.1 was used to test potential recombination events. Bootscan analysis in the SimPlot package version 3.5.1 was used to test potential recombination events. Similarity was calculated in each window of 200 bp by the Kimura (2-parameter) distance model with a transition-transversion ratio of 2.0. The window was successively advanced along the genome alignment in 20 bp increments. For bootscan analysis, the neighbor-joining algorithm was run with 1000 bootstrap replicates. A threshold of 70% or more of the observed permuted trees indicated potential recombination events. Potential genomic components were identified based on genetic distances and phylogenetic analyses.

### Amino acid analysis

SH2016 ORFs were compared to 5 prototype sequences and the 38 remaining HAdV-C whole genome sequences from GenBank. The complete genome of SH2016 strain was annotated using AC_000017 (HAdV-1 prototype strain) as template.

### Ethics statement

This study was reviewed and approved by the human Research Ethics Committee Ethics Review Committee of the Shanghai Public Health Clinical Center. All methods used in this study were performed in accordance with the relevant guidelines. Written informed consent for the collection of throat swabs for pathogenic identification was obtained from the participants involved in this study.

## Supplementary information

Supplementary Information
