# Supplementary material for: Genome Analysis of A Novel Recombinant Human Adenovirus Type 1 in China
Source: Sci Rep. 2019 Mar 12;9:4298. doi: 10.1038/s41598-018-37756-4 (PMC6414723; doi:10.1038/s41598-018-37756-4)
Supplement: Supplementary file 1 — Supplementary Information [file 41598_2018_37756_MOESM1_ESM.pdf]

Genome Analysis of A Novel Recombinant Human Adenovirus Type 1 in China.

Wanju Zhang[1, *], Lisu Huang[2,*]

[1]Shanghai Public Health Clinical Center, Fudan university, Shanghai, 201508, China.
[2]Xinhua Hospital affiliated Shanghai Jiaotong University, Shanghai, 200092, China
* These authors contributed equally to this work.

Supplementary Information

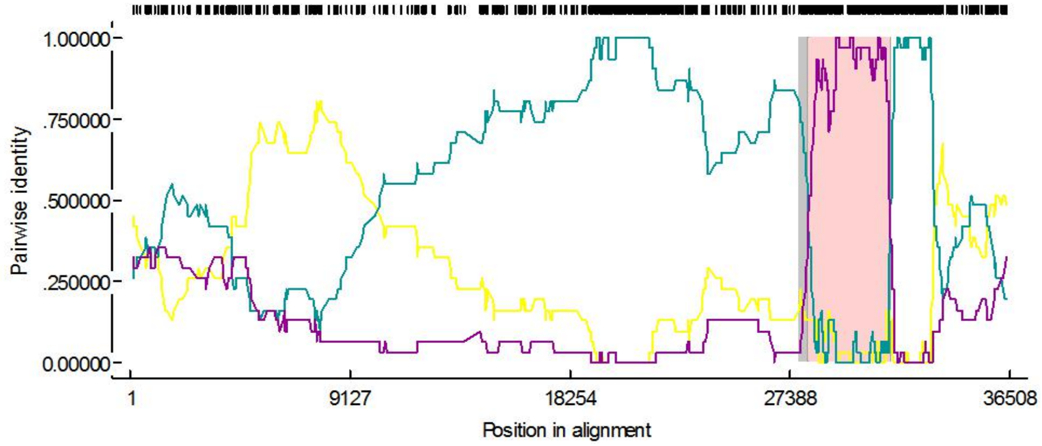

Supplemental Figure 1: Recombinant SH2016 RDP Result by RDP4. Yellow line: JX173083-KX384959 (minor parent), cyan line: KX384959 (major parent), purple line: JX173083 (minor recombinant parent)

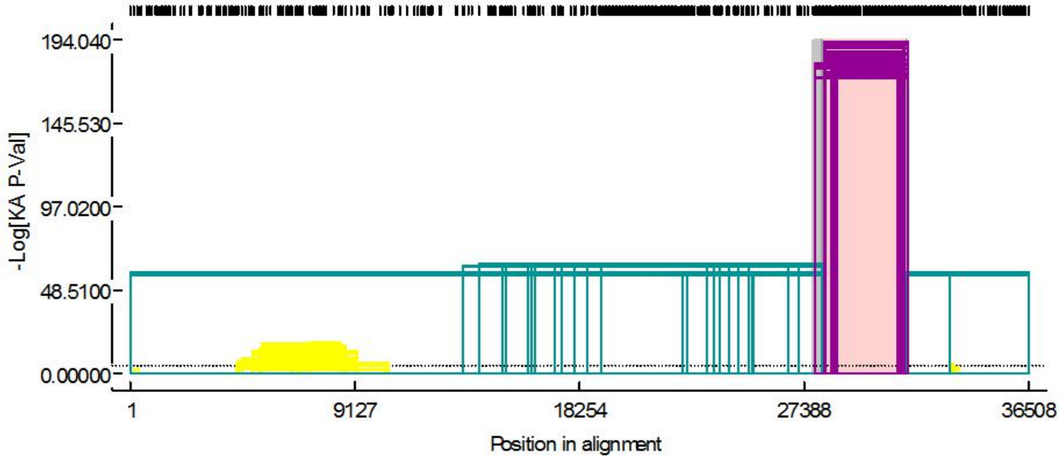

Supplemental Figure 2: Recombinant SH2016 GENECONV Result by RDP4. Yellow line: JX173083-KX384959 (minor parent), cyan line: KX384959 (major parent), purple line: JX173083 (minor recombinant parent)

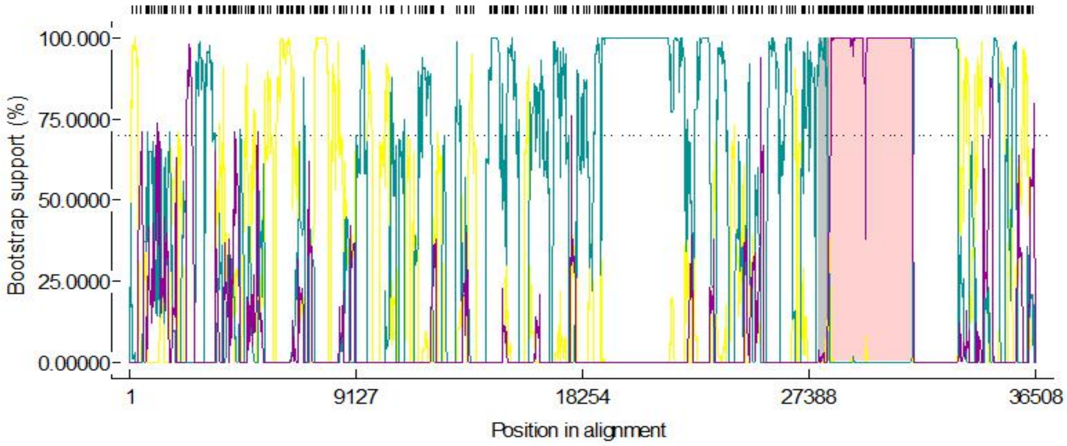

Supplemental Figure 3: Recombinant SH2016 Bootscan Result by RDP4. Yellow line: JX173083-KX384959 (minor parent), cyan line: KX384959 (major parent), purple line: JX173083 (minor recombinant parent)

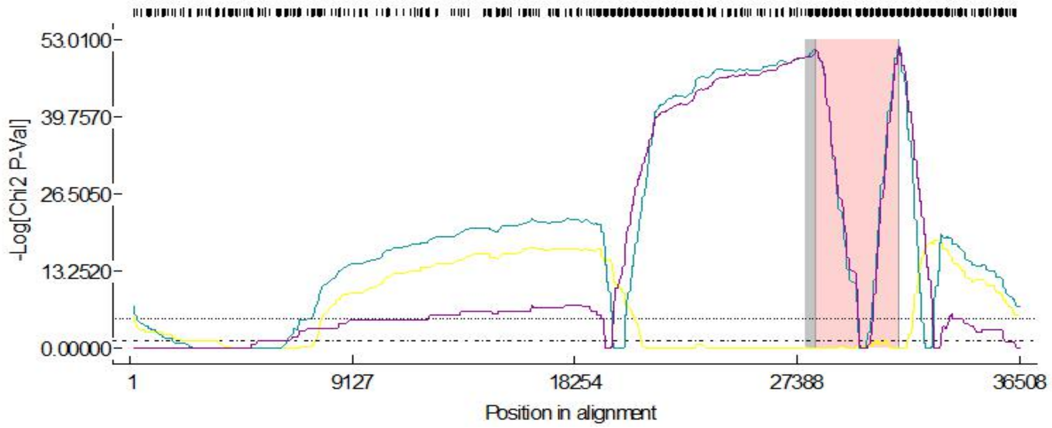

Supplemental Figure 4: Recombinant SH2016 MaxChi Result by RDP4. Yellow line: JX173083-KX384959 (minor parent), cyan line: KX384959 (major parent), purple line: JX173083 (minor recombinant parent)

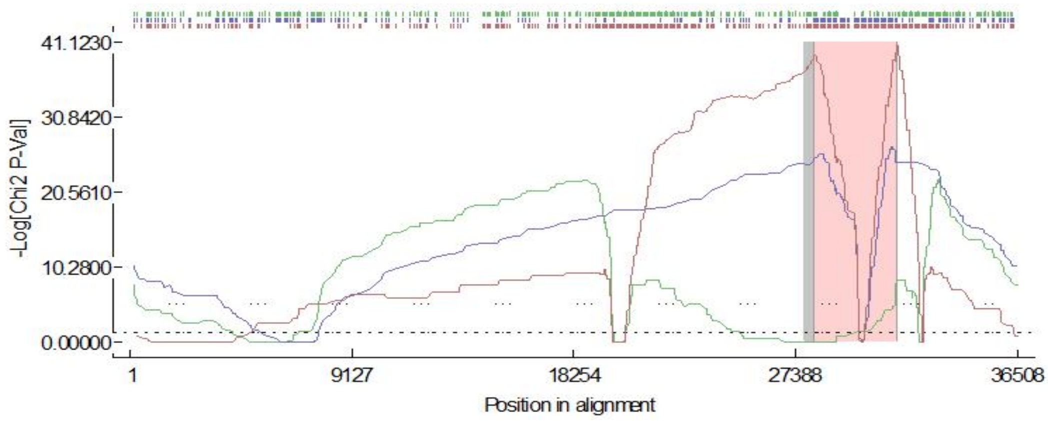

Supplemental Figure 5: Recombinant SH2016 Chimaera Result by RDP4. Cyan line: KX384959, purple line: SH2016, Light blue line: JX173083.

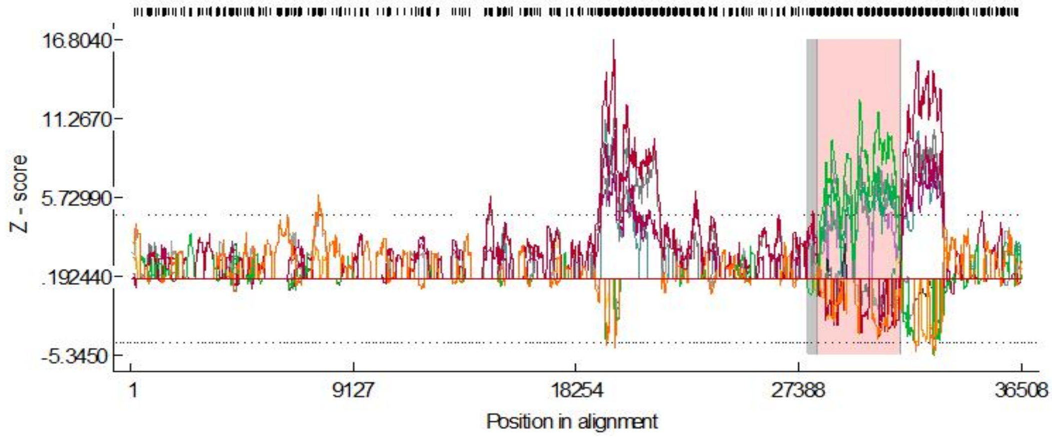

Supplemental Figure 6: Recombinant SH2016 SiScan Result by RDP4. Yellow line: JX173083-KX384959 (minor parent), cyan line: KX384959 (major parent), purple line: JX173083 (minor recombinant parent)

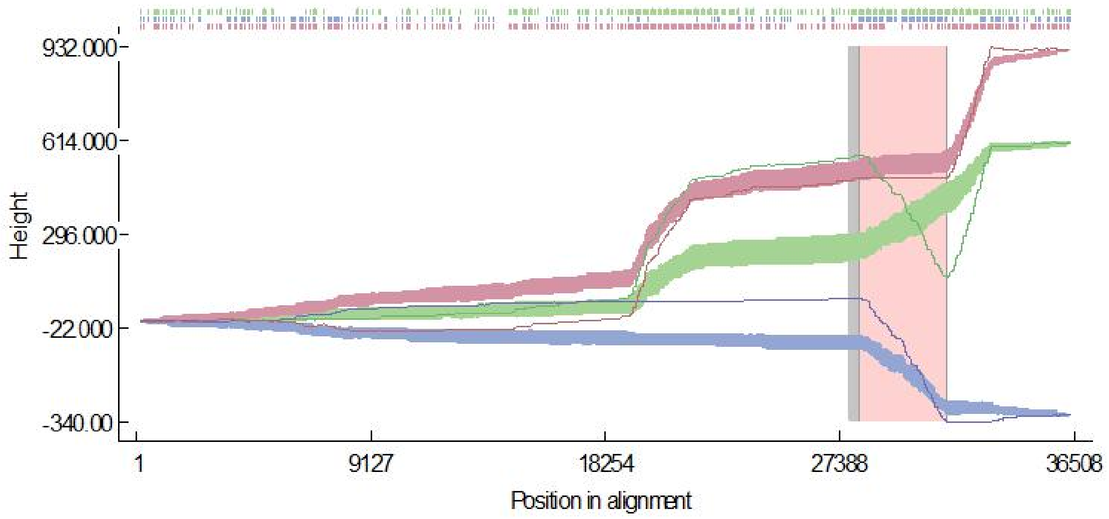

Supplemental Figure 7: Recombinant SH2016 3Seq Result by RDP4. Cyan line: KX384959, purple line: SH2016, Light blue line: JX173083.

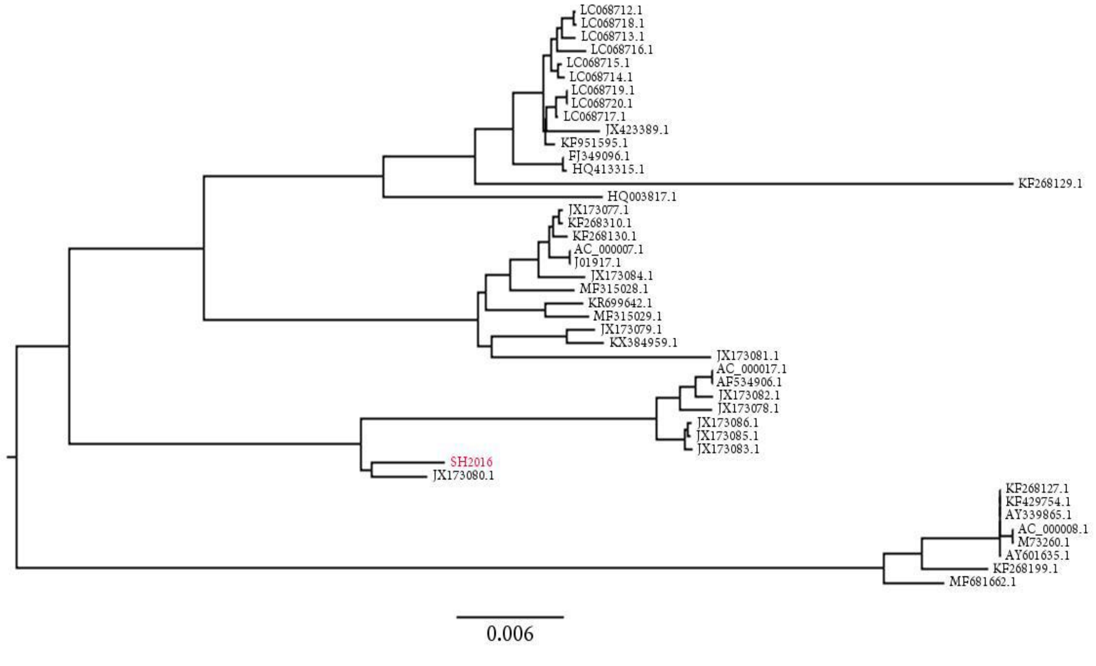

Supplemental Figure 8: Mid-point rooted phylogenetic tree based on the nucleotide sequences of **whole genome** from representative members of the HAdV-C group. Phylogenetic tree was reconstructed by the maximum likelihood (ML) method implemented in IQ-TREE 1.6.7.1 under the GTR+I+G nucleotide substitution model, which was selected by jModeltest. Support for the inferred ML tree was inferred by bootstrapping with 1000 replicates.

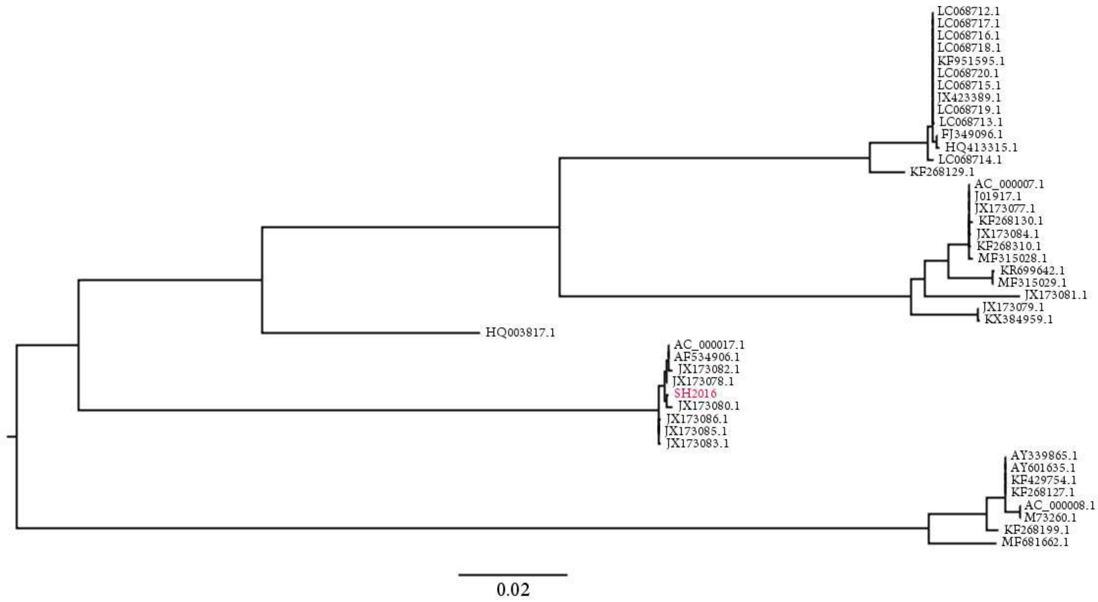

Supplemental Figure 9: Mid-point rooted phylogenetic tree based on the nucleotide sequences of **hexon gene** from representative members of the HAdV-C group. Phylogenetic tree was reconstructed by the maximum likelihood (ML) method implemented in IQ-TREE 1.6.7.1 under the GTR+I+G nucleotide substitution model, which was selected by jModeltest. Support for the inferred ML tree was inferred by bootstrapping with 1000 replicates.

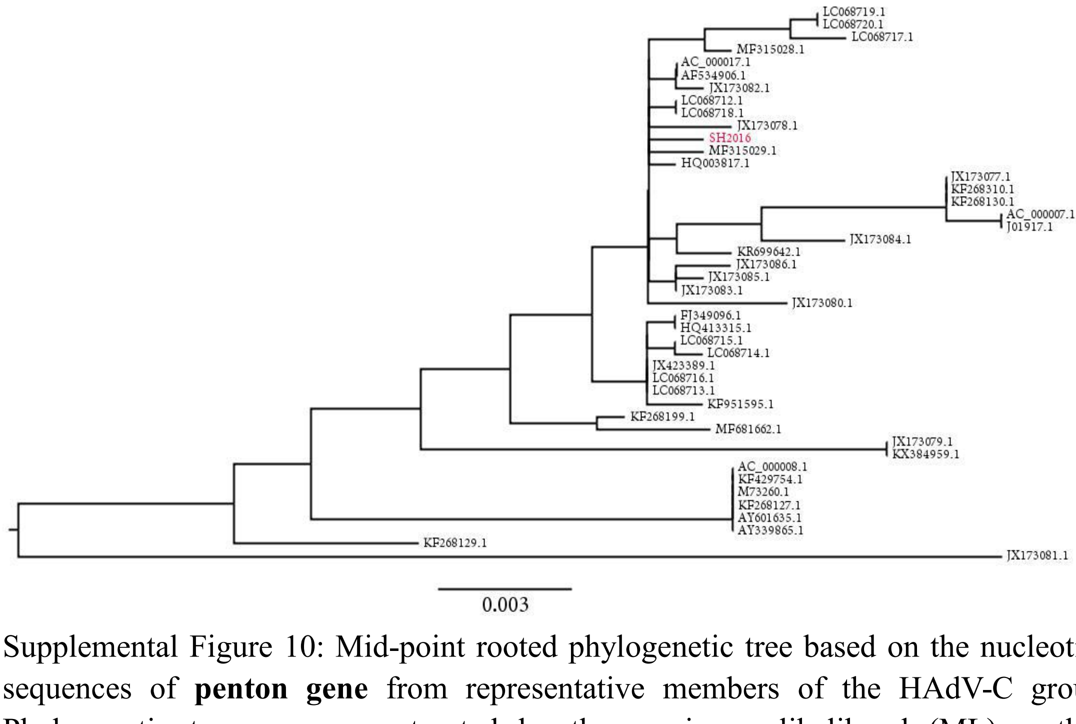

Supplemental Figure 10: Mid-point rooted phylogenetic tree based on the nucleotide sequences of **penton gene** from representative members of the HAdV-C group. Phylogenetic tree was reconstructed by the maximum likelihood (ML) method implemented in IQ-TREE 1.6.7.1 under the GTR+I+G nucleotide substitution model, which was selected by jModeltest. Support for the inferred ML tree was inferred by bootstrapping with 1000 replicates.

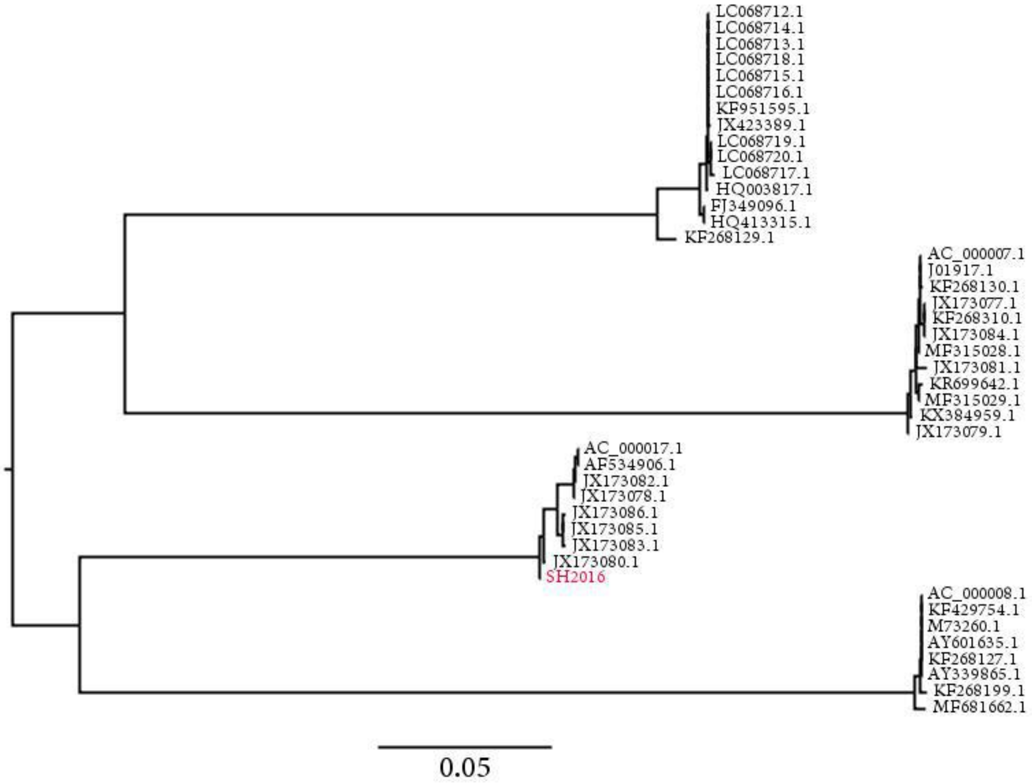

Supplemental Figure 11: Mid-point rooted phylogenetic tree based on the nucleotide sequences of **fiber gene** from representative members of the HAdV-C group. Phylogenetic tree was reconstructed by the maximum likelihood (ML) method implemented in IQ-TREE 1.6.7.1 under the TPM2uf+I nucleotide substitution model, which was selected by jModeltest. Support for the inferred ML tree was inferred by bootstrapping with 1000 replicates.

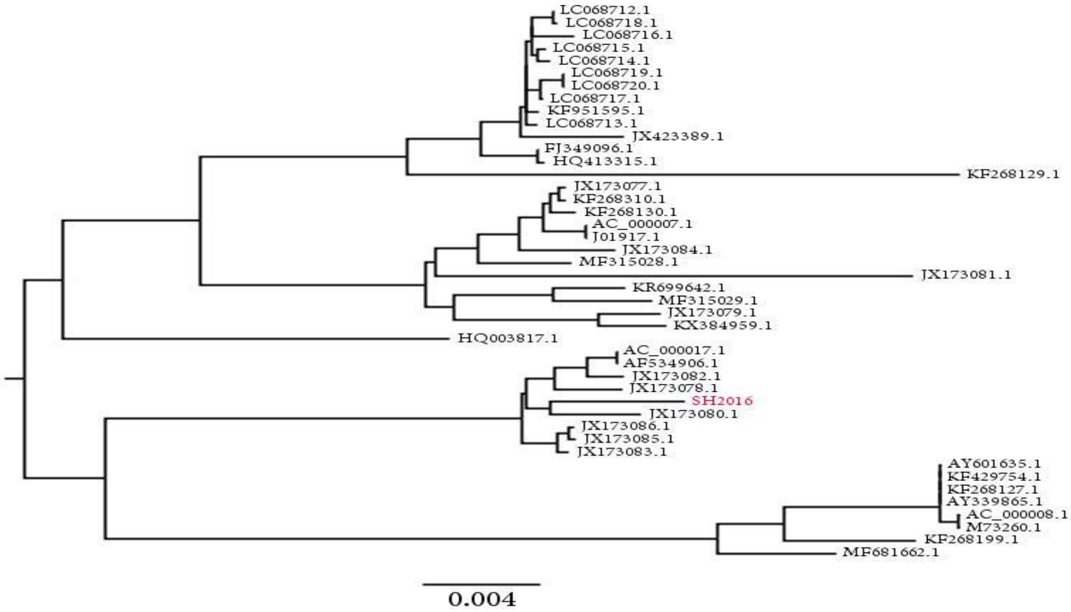

Supplemental Figure 12: Mid-point rooted phylogenetic tree based on the nucleotide sequences of **the left region of recombinant point** from representative members of the HAdV-C group. Phylogenetic tree was reconstructed by the maximum likelihood (ML) method implemented in IQ-TREE 1.6.7.1 under the GTR+I+G nucleotide substitution model, which were selected by jModeltest. Support for the inferred ML tree was inferred by bootstrapping with 1000 replicates.

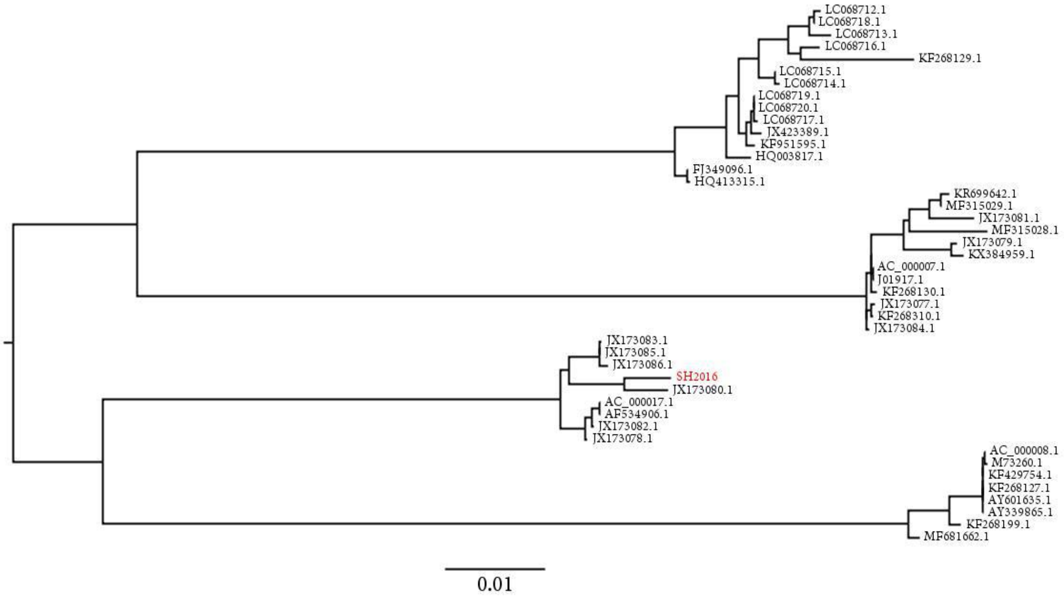

Supplemental Figure 13: Mid-point rooted phylogenetic tree based on the nucleotide sequences of **the right region of recombinant point** of SH2016 strain from representative members of the HAdV-C group. Phylogenetic tree was reconstructed by the maximum likelihood (ML) method implemented in IQ-TREE 1.6.7.1 under the TVM+I+G nucleotide substitution model, which were selected by jModeltest. Support for the inferred ML tree was inferred by bootstrapping with 1000 replicates.

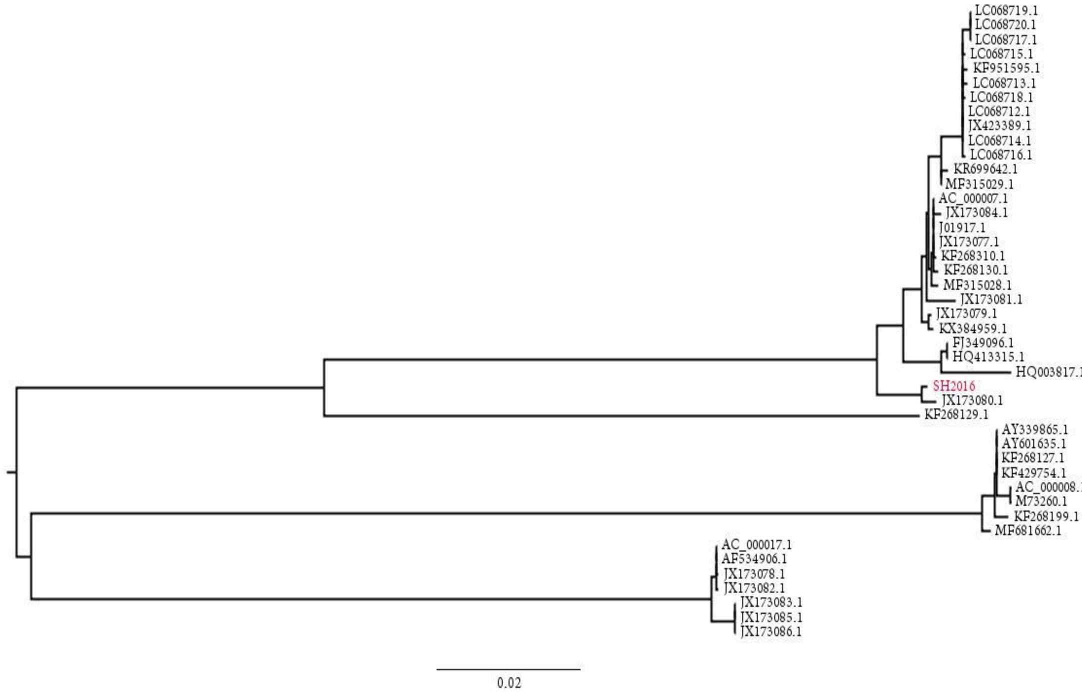

Supplemental Figure 14: Mid-point rooted phylogenetic tree based on the nucleotide sequences of **the recombinant region** of SH2016 strain from representative members of the HAdV-C group. Phylogenetic tree was reconstructed by the maximum likelihood (ML) method implemented in IQ-TREE 1.6.7.1 under the GTR+G nucleotide substitution model, which was selected by jModeltest. Support for the inferred ML tree was inferred by bootstrapping with 1000 replicates.
